# Identification and evaluation of probiotic potential of *Bifidobacterium breve* AHC3 isolated from chicken intestines and its effect on necrotizing enterocolitis (NEC) in newborn SD rats

**DOI:** 10.1371/journal.pone.0287799

**Published:** 2023-11-02

**Authors:** Xiaopei Lin, Changjun Wu

**Affiliations:** 1 Department of Pediatrics, Maternity and Child Health Care Hospital Affiliated to Anhui Medical University (Anhui Maternity and Child Health Care Hospital), Hefei, Anhui, China; 2 Institute of Microbiology, Anhui Academy of Medical Sciences, Hefei, Anhui, China; Zagazig University Faculty of Veterinary Medicine, EGYPT

## Abstract

Necrotizing enterocolitis (NEC) is a severe intestinal disease of the newborn infants, associated with high morbidity and mortality. It has been reported that *Bifidobacterium* could protect the intestinal barrier function and reduce the risk of NEC. This study aimed to evaluate the probiotic potential of *Bifidobacterium* strains isolated from the chicken intestines and its effect on necrotizing enterocolitis in newborn SD rats. Out of 32 isolates, *B*. *breve* AHC3 not only exhibited excellent probiotic potential, including tolerance to artificial simulated gastric conditions, adhesion to HT-29 cells, antioxidant capacity and antibacterial activity, but also possessed reliable safety. Additionally, NEC model was established to further investigate the effect of *B*. *breve* AHC3 on necrotizing enterocolitis in newborn SD rats. It was illustrated that administration of *B*. *breve* AHC3 significantly not only reduced the incidence of NEC (from 81.25% to 34.38%) (*P*< 0.05), but also alleviated the severity of ileal injury (*P*< 0.05). Compared with NEC model, *B*. *breve* AHC3 could significantly decrease the level of proinflammatory factor TNF-α (*P*< 0.05) and increase the level of antiinflammatory factor IL-10 (*P*< 0.05) in the ileum of NEC rats. Through the intervention of *B*. *breve* AHC3, the gray value of inducible nitric oxide synthase (iNOS) in intestinal tissue of NEC rats was significantly reduced (*P*< 0.05). It was indicated that *B*. *breve* AHC3 exhibited prominent probiotic potential and reliable safety. In the neonatal SD rat model of NEC, *B*. *breve* AHC3 had an available protective effect on the intestinal injury of NEC, which might be related to reducing the inflammatory reaction in the ileum and inhibiting the expression of iNOS in intestinal tissue cells. *B*. *breve* AHC3 could be used as a potential treatment for human NEC.

## Introduction

Necrotizing enterocolitis is the most common and serious intestinal disease with fatal risk in newborns, especially premature infants, which is seriously harmful to the health and life of newborns [1, 2]. NEC usually represents symptoms of anorexia, weight loss, abdominal distension and hematochezia in clinic, and the main pathological manifestation of NEC is inflammatory hemorrhagic necrosis of intestines [3, 4]. It is reported that the number of newborns diagnosed with NEC accounts for 2%-5% of the total number of newborns in the intensive care unit, the mortality rate is 15%-30% of the confirmed cases, and the mortality rate of newborns requiring surgical intervention is even higher [5–7]. In addition, the inevitable complications, including short bowel syndrome, heterotopic disease and intestinal stenosis, seriously interfere with the quality of life of infant suffering from NEC [8, 9]. The pathogenesis of NEC is complex and multifactorial [10]. Although decades of research have been carried out on NEC, its pathogenesis is still unclear, and there is still lacking of effective prevention and treatment measures. Evidence suggests that the main risk factors resulting in NEC consist of premature delivery, enteral nutrition, pathogenic bacterial colonization, hypoxia and intestinal ischemia [1, 11].

It has been proved that risk factors of NEC could induce inflammatory cascade reaction [12]. Excessive intestinal inflammation plays an important part in the pathogenesis of NEC. Cytokine is the key regulator of inflammation during NEC [13]. The severity of cytokine production is considered as a biomarker of intestinal injury. Tumor necrosis factor-α (TNF-α) and Interleukin-10 (IL-10) are regard as a crucial diagnostic value in NEC [14]. TNF-α, as a significant proinflammatory cytokine, is closely related to intestinal inflammation and NEC [15]. A series of cytokines such as interleukin-1 (IL-1), IL-6, IL-8, etc. produced through cascade reaction are caused by TNF-α, the transcription and expression of inflammatory genes are triggered, leading to excessive inflammatory cascade reaction [9, 16]. Subsequently, the increase of TNF-α level aggravates the intestinal mucosal injury [17]. It has been confirmed by experiment that the level of TNF-α in infant suffering from NEC increases [18]. IL-10, belonging to the kind of antiinflammatory cytokine, is initially described as an inhibitor on cytokine secretion, which probably has a protective effect on NEC by inhibiting inflammatory reaction. IL-10 plays an antiinflammatory part in the immune system to place restriction on the extent of immune response [19]. Study shows that spontaneous enterocolitis occurs in mice lacking of IL-10 [20]. On the contrary, application of IL-10 can reduce inflammatory reaction in a mouse model of intestinal ischemia/reperfusion [21]. The inflammatory cascade reaction is triggered by risk factors of NEC, which in turn results in the excessive production of iNOS [22]. It has been suggested that acute NEC in newborn is in connection with increased expression of iNOS and nitrosation [23]. Therefore, searching for new effective drugs to help treating and preventing the development of NEC will become a more popular trend at present.

Probiotic is defined as a group of living and non-pathogenic microorganisms colonizing in the intestine and providing benefits to the host [24]. *Bifidobacterium* is one of the most commonly used probiotic in clinical treatment presently [25]. *Bifidobacterium* is Gram-positive, anaerobic, polymorphous, non-motile and non-spore forming, which includes more than 30 species currently [26]. It is dominant in the intestinal flora of normal newborns [27]. *Bifidobacterium* is beneficial to many aspects of human health through different ways, mainly including enhancement of intestinal barrier, regulation of immune system, inhibition on pathogens, nutrition supplement as well as reinforcement and expansion of host metabolism [28, 29]. Specific probiotics, especially *Bifidobacterium*, have been proved to protect intestinal integrity, maintain intestinal barrier function and have beneficial effects on NEC [13, 30]. Khailova et al. found that oral treatment of *B*.*bifidum* could prevent the intestine from disease in the NEC model [13]. Underwood et al. observed that administration of *B*. *longum* subsp. *infantis* had protective effect on NEC and reduced NEC-related inflammation [31]. It was documented by Wu et al. that *B*. *adolescentis* significantly reduced the incidence of intestinal injury and prevented the morbidity of NEC in premature newborn rats [32]. Therefore, biological exploration of new strains with probiotic potential, especially beneficial effect on NEC, has become a hot spot of current research on prevention and treatment of NEC.

The chicken gastrointestinal microflora has attracted more and more attention in recent years due to its biodiversity [33]. The avian gastrointestinal microflora is composed of tens of billions of microbes, which contributes to digestion and absorption of nutrients, immune system development and pathogen inhibition [34]. It plays an important role in maintaining the normal and harmonious functions in the whole organism. In the chicken gastrointestinal microflora, approximately 900 species of microbes identified belong to 117 genera [35]. It has been reported that the content of *Bifidobacterium* in poultry cecum is up to 10^9^−10^10^ CFU/g [36]. Therefore, the chicken gastrointestinal tract is regarded as an excellent area for screening more strains with significant probiotic characteristics. Aalipanah et al. reported that a strain of *B*.*bifidum* isolated from the chicken intestine showed potential probiotic characterization [37]. A strain of *B*. *dentium* from chicken intestines was isolated by Palacios et al., exhibiting the highest phytase activity [38]. Collado et al. also isolated 4 *B*. *longum* strains from chicken intestines, it was indicated that the adhesion of *Bifidobacterium* strains originated from animal was significantly better than that of *Bifidobacterium* strains from human [39].

Hefei free-ranged chicken is an excellent local traditional breed of chicken originating from the central and western regions of Anhui, China. It belongs to the flaxenfeather chicken and has a long growth period. The chicken mainly lives in hilly areas and is limited to scattered breeding by local farmers currently, which has strong foraging ability and mainly feeds on vegetable leaves, grass seeds, leaves, and small insects. However, it has so far been rare reports on the isolation, identification, and probiotic potential of *Bifidobacteria* in the gastrointestinal tract of Hefei free-ranged chicken, in despite of a significant increase in literature on the screening and probiotic potential of probiotics in the chicken gastrointestinal tract in the past decade.

The purpose of the present study was two folds: first, to evaluate the probiotic potential of *Bifidobacterium* isolated from the free-ranged chicken intestines *in vitro*, mainly including tolerance to artificial simulated gastrointestinal conditions, adhesion ability to HT-29 cells, antioxidant capacity and antibacterial activity. Second, a NEC model was established by hypoxia, cold stimulation, artificial feeding and LPS induction to further investigate the effect of *B*. *breve* AHC3 on necrotizing enterocolitis in newborn SD rats *in vivo*, through observing incidence rate of NEC, severity of ileal injury, level of inflammatory factors and expression of iNOS, to provide theoretical basis for clinical application of *B*. *breve* AHC3 to prevent and treat NEC.

## Materials and methods

### Isolation, purification and preliminary identification of *Bifidobacterium* spp.

The ilea and caeca of 10 healthy adult free-ranged chickens (*Gallus gallus*) were collected from peasant households in Hefei(E 116°42′19′′, N 31°46′25′′), Anhui Province, China and immediately transported to the laboratory under sterile and coolingconditions. The intestineswere cut open with sterile scissors, the inner wall of the intestine was rinsed with sterile normal saline to remove the contents of the intestine. Onegramof intestinal mucosa was scraped and put into 9 mLPBS containing 0.05% L-cysteine hydrochloride (Sigma Aldrich, USA)(PBSc) for tenfold serial dilutions, subsequently. A volume of 0.1mL sample solutionwasspreadon selective de Man, Rogosa, and Sharpe (MRS, HopeBio, China) platescontaining 0.05% (w/v) L-cysteine hydrochloride(MRSc) plus 100 mg/L mupirocin (HopeBio, China), and placed in anaerobic environment for culture at 37°C for 72 h. Selection and purification the single white colonies by streaking on selective MRSc plate. Observationon colony morphological characteristics, Gram staining,catalase production and biochemical experiments were included in the preliminary identification on isolates. All the experiments in this study have been carried out in triplicates. The commercial strain *Bifidobacterium animal* subsp. *Lactis* BB-12 (BB-12) with good probiotic characteristics was selected as the reference strain, which was provided by the Institute of Microbiology, Anhui Academy of Medical Sciences, China.

### Evaluation of probiotic potential of *Bifidobacterium* spp.

#### Tolerance to artificial simulated gastrointestinal conditions

According to the method described previously, tolerance to simulated gastrointestinal conditionsof isolated *Bifidobacterium* strains was determined with simple modifications [40]. The isolated strains were anaerobically cultured in MRSc broth supplemented with 0.1% (w/v) ascorbic acid at 37°C for 48 h, centrifugation by 10000 × *g* for 10 min at 4°C and the particles were washed twice with PBSc buffer (pH = 7.4). Using 4 mL of artificial simulated gastric juice (125 mM NaCl, 7 mM KCl, 45 mM NaHCO_3_, 3 g/L pepsin (Shanghai Lanji Technology Development Co., Ltd.), pH = 2.0 and sterilization by filtration) to suspend the particles (10^8^CFU/mL), fowlled byincubation in 37°C water bath for 3 h. After tenfold serial dilutions, 0.1 mL suspension was spread onMRScplate, and the number of viable cells was measured by colony count. After treatment with artificial simulated gastric juice for 3 h, the isolateswere obtained under centrifugation by 10000 × *g* for 10 min at 4°C and the particles were washed twice with PBSc buffer. Using 4 mL of artificial simulated intestinal juice (22 mM NaCl, 3.2 mMKCl, 7.6 mM NaHCO_3_, 1 g/L trypsin (Shanghai Lanji Technology Development Co., Ltd.), 0.3 g/L bile oxgall (Beijing Solarbio Science and Technology Co., Ltd.), pH = 8.0 and sterilization by filtration) to suspend the particles, fowlled by incubation in 37°C water bath for 5 h.After tenfold serial dilutions, 0.1 mL suspension was spread on MRSc plate, and the number of viable cells was measured by colony count.

#### Adhesionto HT-29 cells

Adhesion of isolated *Bifidobacterium* strains to HT-29 cells was evaluated as the method described by Lee et al. [41] with slightmodifications. HT-29 cells (Hunan Fenghui Biotechnology Co., Ltd.) were cultivated in Dolbecco’sModified Eagle Medium F-12 (DMEM/F12, Gibco, USA)culture supplemented with 10% (v/v) fetal bovine serum (ZhengjiangTianhang Biotechnology Co., Ltd.) in a 5% CO_2_ incubator at 37°C for 48 h. The cells were harvested and added into sterile 24-well plate (10^6^cells/well), followed by cultivation for 48 h. The culture was refreshed every 24 h. Added 0.5 mL of *Bifidobacterium* strains (10^8^ CFU/mL) into the wells and incubated the mixture at37°C for 2 h anaerobically. The wells were washedfor 3 times with PBSc solution, then a drop of Triton X-100 (Biofoxx, Germany) was added into each well, and the lysates were subsequently spread on MRSc plates. The number of *Bifidobacterium* was measured by colony count.

#### Antioxidant capacity

The radical scavenging activity of 2, 2-diphenyl-1-picrylhydrazyl (DPPH) was detected using the procedure [42] with minor modifications. In brief, 0.2 mLPBS containing 10^9^ CFU/mL *Bifidobacterium* was added into 1 mL DPPH methanol solution (100 μM). After exposed in the dark at 37°C for 20 min, the mixturewas centrifugated at 8000 × *g* for 5 min and the absorbance value at 517 nm was measured. According to the methodology used [40], the radical scavenging activity of 2, 2-azinodi-[3-ethylbenzthiazoline sulfonate] (ABTS) was investigatedwith simple modifications. A volume of 0.1 mL PBS containing 10^9^ CFU/mL *Bifidobacterium* was added into 1 mLABTS working solution (7 mM ABTS solution, 2.45 mM potassium persulfate solution and incubation in the dark at 37°C for 12 h). After exposed in the dark at 37°C for 20 min, the mixture was centrifugated at 8000 × *g* for 5 min and the absorbance value at 734 nm was measured. The radical scavenging activity of superoxide anion was assayedas the method described previously [43] with slight modifications. A volume of 0.5 mL PBS containing 10^9^ CFU/mL *Bifidobacterium* was added into 1.5 mL Tris-HCl solution (pH = 8). After placed at 25°C for 20 min, 0.2 mL of pyrogallol solution (25 mM) was added into the mixture. After exposed in the dark for 5 min, the reaction was terminated with 0.25 mL hydrochloric acid. The mixture was centrifugated at 8000 × *g* for 5 min and the absorbance value at 325 nm was measured.

#### Antibacterial activity

Antibacterial activity of *Bifidobacterium* strains was performedwith the protocol described by Piyadeatsoontorn et al. [44] with some modifications. In this research, *Escherichia coli* ATCC8099, *Staphylococcus aureus* ATCC6538, *Salmonella enterica* ATCC9120 and *Shigellasonnei* BNCC192105 were applied as indicator strains. First, 0.1mL fresh indicator bacteria culture (10^7^ CFU/mL) was spread on LB plate. After the plate surface was air-dryin super-clean worktable, the sterile discswere placed on the plate. A drop of cell free supernatant (CFS) of *Bifidobacterium* was added onto the disc, and the plates were incubated at 37°C for 24 h. The diameter of inhibition circle was measured byvernier caliper to determine the antibacterial activity of *Bifidobacterium* strains.

### Evaluation of safety

#### Hemolysis test

Hemolysis test was evaluated according to the method detailed by Menezes et al. [45]. *Bifidobacterium* strain was streaked on Columbia agar plate containing 5% sheep blood and the hemolytic result was observed after incubation for 24 h. Presence of hydrolytic circle (β-hemolysis) was classified as a positive result. Presence of green circle (α-hemolysis) or no hydrolytic circle (γ-hemolysis) was classified as non- hemolysis.

#### Antibiotic sensitivity assay

Antibiotic sensitivity was measured by the method used [46] with slight modifications. A volume of 0.1 mL fresh *Bifidobacterium* culture (10^7^ CFU/mL) was spread on MRSc plate. After the plate surface was air-dryin super-clean worktable, the antibiotic discswere placed on the plate with sterile tweezer, and the plates were incubated at 37°C for 24 h. The diameter of the inhibition zone was measured with a vernier caliper to determine its antibiotic sensitivity, and the results were classified according to the microbiological breakpoints for antimicrobials issued [47].

#### Cytotoxicity

The cytotoxicity of *Bifidobacterium* strain to HT-29 cells was conducted by 3-(4,5-dimethylthiazol-2-yl)-2,5-diphenyltetrazolium bromide (MTT)method with slight modifications [48]. In short, HT-29 cells were addedinto sterile 24-well plate (10^4^ cells/well) containingDMEM/F12 medium and 10% fetal bovine serum, and incubated in a 5% CO_2_ incubator at 37°C for 24 h. Subsequently,a volume of 200 μL CFS of *Bifidobacterium* strain or PBSc solution was added and further incubated at 37°C for 24 h.The wells were washed with PBScsolution and treated with 200 mL MTT solution (0.5 mg/mL) for 3.5 h.The absorbance value at 540 nm was measured.

#### Biogenic amines production

The production of biogenic amines was detected as protocol described previously [49]. Pyridoxal-5-phosphate (0.005%) as a cofactor in the decarboxylation reaction was added to the MRSc medium (pH = 5.3). The *Bifidobacterium* strain was streaked on the MRSc plate supplemented with amino acids (lysine, histidine, arginine, tyrosine and ornithine) (Beijing Solarbio Science and Technology Co., Ltd.), and the final concentration was 0.5%. Subsequently, the plate was incubated at 37°C for 72 h. The positive result was confirmed through the color changed from yellow to purple of the indicator bromocresol violet.

#### 16s rRNA gene sequencing for molecular identification

The isolated *Bifidobacterium* strain was identified by 16S rRNA gene at the species level. Genomic DNA was extracted and used for PCR amplification [44]. Briefly, the 16S rRNA gene was amplified by using the primer pair 27f (5 ’-AGAGTTTGATCCTGGCTCAG -3’) and 1492r (5 ’- CTACGGCTACCTTGTTACGA -3’). Total 50μL system as follows: 1.0μL template DNA, 1.0μLTaq DNA polymer, 5.0μL 10 × PCR buffer, 1.0μL10 mM dNTP, 1.5μL 10μM upstream and downstream primers,respectively, 39.0μL ddH_2_O, andthermal cycle parameters performed as follows: initial denaturation at 95°C for 300 s, 35 denaturation cycles at 95°C for 30 s, annealing at 58°C for 30 s, extension at 72°C for 90 s, and final extension at 72°C for 420 s for PCR reaction. A volume of 3 μL PCR amplification fragment was visualized at100 V for1 h by using 1% agarose for electrophoresis. The PCR product was purified and sequenced by Shanghai personal Gene Technology Co., Ltd. 16S rRNA gene sequences were compared and matched with available sequences in NCBI GenBank using BLAST. The homology of the target gene sequence was analyzed, and the phylogenetic tree was constructed with MEGA 7.0, based on the bootstrap value of 1000 replications. The identified sequence was uploaded to NCBI gene bank database.

#### Scanning electron microscope (SEM) observation

In order to observe the morphology of isolated *Bifidobacterium* strain exhibiting probiotic potential, the strain was incubated at 37°C for 18 h in MRSc broth, and the culture was observed under a scanning electron microscope by using critical point drying (CPD) method [50].

#### Ethics statement

The protocol was approved by the experimental animal ethics committee of Anhui Academy of Medical Sciences. According to the procedures approved by the Institutional Animal Care and Use (IACUC), euthanasia must be carried out in rats when the animal meets one of the following criteria: (1) loss of appetite and significant weight loss, (2) sharp decrease in activity, loss of sensitivity to external stimuli, pale and cyanotic skin, and no response to treatment, (3) poor prognosis and moribund state. Daily inspections were conducted by professionals to assess the health status of rats. At the end of the experiment or one of the above criteria, euthanasia to rats were carried out by decapition to alleviate the pain of the animals, and the number of rats used was minimized as much as possible based on the completion of the experiment. Throughout the modeling process, no surgical procedure was performed. After conducting hypoxic cold stress experiments, timely oxygenation treatment and rapid warming to rats were carried out to reduce discomfort in the animals. In addition, the rats were treated as gentle as possible during the experimental operation and comforted as much as possible after the experiment.

#### Establishment of the NEC model

128 newborn Sprague-Dawley rats without specific pathogen within 2 h after birth were purchased from Laifu Animal Breeding Farm, Nanjing, China(SPF grade). 128 SD rats were randomly divided into 4 groups. Group A was the blank control group (n = 32), Group B was *Bifidobacterium* control group (n = 32); Group C was NEC model group (n = 32), and group D was *Bifidobacterium* intervention group (n = 32). Group A and B were fed by female rats, Group C and D were fed with artificial formula milk powder every 4 h. The rats were given 0.1 mL by gavage for the first time, 0.1mL increased the next day, and the maximum amount was no more than 0.3 mL.The NEC model of neonatal SD rats was established by hypoxia-cold stress, artificial formula feeding and LPS (Sigma, USA) treatment according to the method as previouslydescribed by Xiang [51] with slight modifications.Group C and D were treated with hypoxic-reoxygenation (first breathing 100% nitrogen for 3 min, then immediately breathing 100% oxygen for 3min), and a cold stress (4°C, 10 min) twice a day for 3 d. Meanwhile, neonatal SD rats were induced NEC by intraperitoneal injection of LPS (5 mg/kg, prepared with normal saline to 5 mg/mL) once a day for 3 d. Group B and D were treated by *Bifidobacterium* gavage after daily modeling stimulation, and *Bifidobacterium* was added to 10 mL normal saline or artificial formula milk (5 × 10^9^ CFU/mL), 0.2 mL a day by gavage for 3 d. Group A and C were treated with the same amount of normal saline instead. Group A and B were not treated with modeling stimulation.

#### Pathological observation on intestinal tissue

A 1 cm of the proximal intestinal tube of ileum was taken out aseptically and fixed in 10% formaldehyde solution, embedded in paraffin, coronal sectioned, and stained with hematoxylin eosin (H & E) to observe the pathological change of the intestine under light microscope.According to the pathological scoring criteria reported [52], the intestinal tissue damage was observed and scored by the blind assessor. It was divided into 4 grades as follows: 0, normal; 1 point, slight separation of submucosa and / or lamina propria; 2 points, moderate separation of submucosa and / or lamina propria, edema of submucosa and/or muscle layer; 3 points: severe separation of submucosa and / or lamina propria, severe edema of submucosa and / or muscle layer, local villi shedding; 4 points: intestinal villi disappeared with intestinal necrosis. Histological score≥2 indicated NEC positive.

#### Determination of TNF-α and IL-10 levels

The levels of TNF-α and IL-10 in intestinal tissue supernatant were investigated by Enzyme-linked immunosorbent assays (ELISA). In short, the intestinal tissue was collected and dissected, the contents of the intestinal cavity were washed with ice physiological saline. After dried with filter paper, the intestinal tissue was homogenized with a homogenizer, followed by centrifugation at 4°C and 10000 r/min for 15 min. Rat TNF-α ELISA detection kit (Shanghai Hufeng Chemical Co., Ltd.) and rat IL-10 ELISA detection kit (Shanghai Hufeng Chemical Co., Ltd.) were used to measure the levels of TNF-α and IL-10 in intestinal tissue according to the manufacturer’s instructions.

#### Detection of iNOS in intestinal tissue

The determination of iNOS in intestinal tissue was carried out by immunohistochemical staining as the methodologyused by Underwood [53]. The terminal ileum tissue was fixed and sectioned conventionally. After deparaffinization and rehydration, the sections were repaired with citric acid buffer (pH = 6.0), and then incubated with 50 μL 0.3% hydrogen peroxide for 10 min at room temperature to block the activity of endogenous peroxidase. The sections were incubated sequentially with 50 μLiNOSprimary antibody for 60 min, 50 μL polymer enhancer (reagent A) for 20 min and 50 μL enzyme labeled anti-rat polymer (reagent B) for 30 min at room temperature. The sections were washed 3 times by PBS after each incubation, followed by visualization with diaminobenzidine (DAB) and hematoxylin counterstaining, respectively. Finally, the sections were sealed. PBS was used as a negative control instead of primary antibody and the specimens were observed under optical microscope. Five different visual fields were randomly selected for observation in each specimen. In the visual field, tan sediment was declared to be a positive reaction, and its density and positive expression site represented the number of antigen distribution and the location of antigen. The average positive staining gray value of iNOS was obtained by image analysis software (Image Pro Plus). The gray value ranged from 0 to 255, and the darker the color of sediment, the larger the measured value.

#### Statistical analysis

Experiments were performed in triplicate in the present research,and results were presentedas mean ± SD. SPSS version 23.0 was employed for data analysis. The variance and differences wereanalyzed by One-way ANOVA with Duncan’s multiple range test and *P* values < 0.05 were considered statistically significant.

## Results

### Preliminary identification on isolated strains

**Fig 1:**
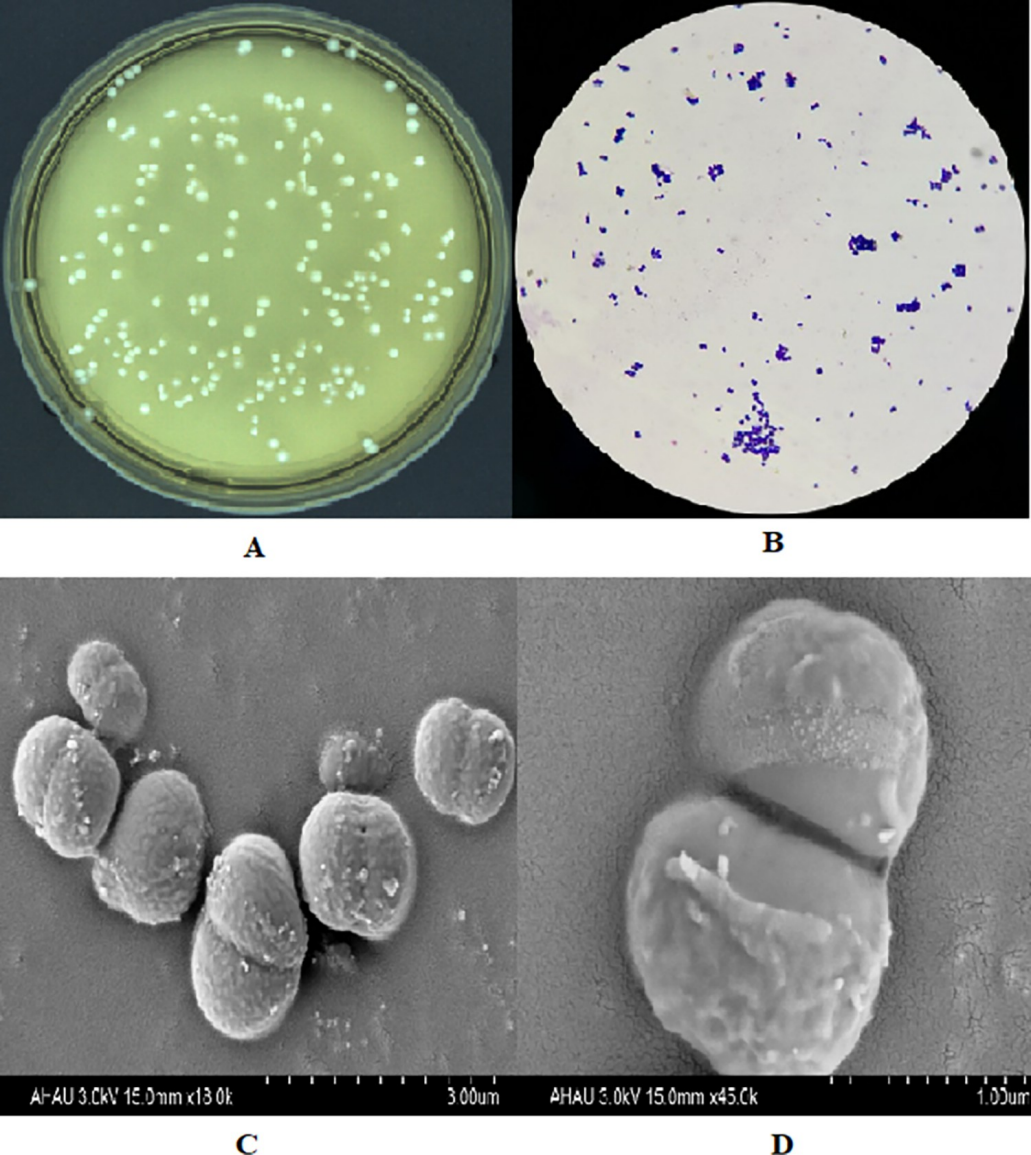
The morphologic characteristics of *B*. *breve* AHC3 strain. (A) Colony morphology of *B*. *breve* AHC3 strain; (B) Gram staining morphology of *B*. *breve* AHC3 strain; (C) SEM image of*B*. *breve* AHC3 strain (18.00k); (D) SEM image of*B*. *breve* AHC3 strain (45.00k).

In the present work, a total of 32 strains were isolated from 10 chicken intestine samples, preliminary physiological and biochemical identification were carried out for 32 isolates by microbiological and biochemical methods. With reference to the physiological and biochemical identification results of *Bifidobacterium* in Berger’s Manual for identification of bacteria, as well as the classification and identification methods for *Bifidobacterium*, 21 isolated strains were preliminarily identified as *Bifidobacterium* spp. (Table 1). All 21 isolates were Gram-positive, and Gram staining morphology of representative strain AHC3 was short rod or ellipsoid shape, appearing in pairs or clusters. Colony morphology of AHC3 was medium-sized or smaller, white or milky white, round, moist, soft, opaque, and neat edges. Colony morphology and Gram staining morphology of representative strain AHC3 were shown in Fig 1A and 1B.

**Table 1:** Physiological and biochemical identification results of 21 isolated strains.

Characteristic	AHC	AHC3
Fructose-6-phosphate	+	+
Gelatin	-	-
Nitrate reduction	-	-
Catalase	-	-
Arginine hydrolysis	-	-
Motile	-	-
Indole	-	-
Arabinose	12	-
Ribose	15	+
Galactose	20	+
Cellobiose	5	+
Esculin	10	-
Fructose	17	+
Gluconate	-	-
Lactose	+	+
Mannitol	3	+
Sorbitol	-	-
Melezitose	5	-
Melibiose	20	+
Raffinose	17	+
Rhamnose	-	-
Salicin	8	-
Glucose	+	+
Sucrose	20	+
Starch	2	-
Trehalose	-	-
Xylose	4	-
Inulin	-	-
Maltose	18	+

AHC: AHC2, AHC5, AHC6, AHC9, AHC10,AHC12, AHC13, AHC15, AHC16, AHC17, AHC19, AHC21, AHC22,AHC24, AHC25, AHC26, AHC27, AHC28, AHC30 and AHC32, 20 isolates.

+,Positive or weakly positive reaction;−,Negative reaction; Number, the number of positive reaction.

### Tolerance to artificial simulated gastrointestinal conditions

Tolerance to artificial simulated gastrointestinal conditions of 21 isolated *Bifidobacterium* strains was determined and the results were seen in Table 2. The survival rates in artificial simulated gastric juice of all 21 *Bifidobacterium* strains declined in various degrees. Out of 21 strains, 16 strains exhibited good tolerance to artificial simulated gastric juice, and the survival rate of AHC28 was the highest with 90.40 ± 4.60%,which showed significant difference with the reference strain BB-12 (75.49 ± 4.60%) (*P*< 0.05). Subsequently, the survival rates in artificial simulated intestinal juice of 14 strains still remained at a relative high level, and AHC28 also displayed the best tolerance with a 79.61 ± 5.65% survival rate. In general, 14 of the 21 isolated strains exhibited high tolerance to artificial simulated gastrointestinal conditions and AHC2, AHC3, AHC6, AHC9, AHC10, AHC12, AHC15, AHC19, AHC21, AHC24, AHC26, AHC27, AHC28and AHC32 were selected for the succedent experiment.

**Table 2:** Tolerance to artificial simulated gastrointestinal conditions of 21 isolated *Bifidobacterium*strains.

Strains	Artificial simulated gastric juice, 3 hsurvival rate (%)	Artificial simulated intestinal juice, 5 h survival rate (%)
AHC2	66.85 ± 4.44^e^	57.13 ± 3.72^bc^
AHC3	76.99 ± 3.35^fg^	65.35 ± 2.30^cdefg^
AHC5	8.97 ± 1.33^a^	-
AHC6	73.66 ± 4.33^efg^	60.43 ± 2.55^bcde^
AHC9	77.99 ± 5.86^fg^	67.52 ± 2.19^defg^
AHC10	82.73 ± 5.67^ghi^	72.96 ± 3.65^ghi^
AHC12	71.82 ± 1.98^ef^	63.20 ± 4.72^cdef^
AHC13	25.68± 3.99^c^	9.05± 2.22^a^
AHC15	65.31 ± 4.60^e^	53.74 ± 3.85^b^
AHC16	16.63 ± 4.87^b^	7.74± 2.59^a^
AHC17	5.06 ± 1.49^a^	-
AHC19	57.67 ± 4.70^d^	51.50± 3.97^b^
AHC21	88.51 ± 2.14^hi^	76.57 ± 4.86^hi^
AHC22	7.02 ± 2.56^a^	-
AHC24	65.05 ± 4.24^e^	54.17 ± 4.60^b^
AHC25	6.53 ± 0.59^a^	-
AHC26	82.47 ± 2.33^ghi^	68.55± 7.44^efgh^
AHC27	66.45 ± 1.08^e^	58.88 ± 3.84^bcd^
AHC28	90.40 ± 4.60^i^	79.61 ±5.65^i^
AHC30	8.64 ± 2.44^a^	-
AHC32	81.32 ± 5.57^gh^	70.40 ± 5.29^fgh^
BB-12	75.49 ± 4.60^fg^	67.28 ± 5.62^defg^

Results are expressed as the mean ± SD;^a-i^, different letters along the column represent statistical significance (*P*< 0.05);-, no bacterial survival detected.

### Adhesionto HT-29 cells

Human colonic carcinoma cell line HT-29 was applied to evaluate the adhesion capacities of 14 *Bifidobacterium* isolates tointestinal mucosa, and the results were presented in Table 3. The adhesion rates of 14 isolates to HT-29 cells ranged from 2.06 ± 0.21% to 25.45 ± 1.63%, AHC12 and AHC28 showed the highest adhesion rate, but there was no significant difference compared with the control strain BB-12(24.98 ± 4.38%)(*P*> 0.05). Six isolates showed lower adhesion ability to HT-29 cell line including AHC2, AHC6, AHC15, AHC19, AHC26, and AHC27 which were not selected to participate in further tests.

**Table 3:** Adhesion capacities to HT-29 cells of 14 isolated *Bifidobacterium* strains.

Strains	Adhesion capacity to HT-29 cells (%)
AHC2	6.95 ± 2.32^a^
AHC3	20.36 ± 1.11^b^
AHC6	2.06 ± 0.21^a^
AHC9	20.15 ± 3.72^b^
AHC10	17.08 ± 1.42^b^
AHC12	25.45 ± 1.63^c^
AHC15	6.92 ± 2.03^a^
AHC19	3.48 ± 1.27^a^
AHC21	17.52 ± 1.98^b^
AHC24	14.97 ± 1.67^b^
AHC26	5.30 ± 1.87^a^
AHC27	6.61 ± 0.56^a^
AHC28	25.10 ± 3.16^c^
AHC32	16.49 ± 1.60^b^
BB-12	24.98 ± 4.38^c^

Results are expressed as the mean ± SD;^a-c^, different letters along the column represent statistical significance (*P*< 0.05).

### Antioxidant capacity

The antioxidant properties of 8 *Bifidobacterium* isolates including AHC3, AHC9, AHC10, AHC12, AHC21, AHC24, AHC28 and AHC32 were detected by measuring the DPPH, ABTS and superoxide anion radical scavenging activities and the results were observed in Table 4. The DPPH radical scavenging activities of 8 *Bifidobacterium* isolates ranged from 30.71 ± 3.72% to 86.82 ± 6.48%, andAHC10 showed the highest DPPH radical scavenging activity(86.82 ± 6.48%), which was higher than BB-12 (80.24 ± 7.99%), but no significant difference was observed (*P*> 0.05). The ABTS radical scavenging activities were in the range of 43.86 ± 4.69% to 90.65 ± 7.28%. AHC10 and AHC3 displayed higher ABTS radical scavenging activities (90.65 ± 7.28% and 88.35 ± 3.19%) than the reference strain BB-12 (87.19 ± 6.65%). Similarly, there was no significant difference (*P*>0.05).Eight *Bifidobacterium* isolateshad varioussuperoxide anion radical scavengingactivities, rangingfrom 4.14 ± 1.21% to 35.01 ± 5.86%, andAHC24 showed the highest superoxide anion radical scavenging activity(35.01 ± 5.86%), which was significantly higher than BB-12 (14.48 ± 4.01%) (*P*<0.05).It was noteworthy that the other 3 *Bifidobacterium* isolates also exhibited significantly higher superoxide anion radical scavenging activity than BB-12, including AHC10 (25.46 ± 4.38%), AHC3 (21.64 ± 2.57%) and AHC28 (20.40 ± 4.08%), respectively. In general, due to the excellent antioxidant capacity of AHC24, AHC10, AHC3 and AHC28, they were selected for the next experiment.

**Table 4:** Antioxidant capacities of 8 isolated *Bifidobacterium* strains.

Strains	Antioxidant properties (%)		
	DPPH scavenging activity	ABTS scavenging activity	Superoxide anion scavenging activity
AHC3	79.32 ± 1.24^c^	88.35 ± 3.19^cd^	21.64 ± 2.57^c^
AHC9	30.71 ± 3.72^a^	43.86 ± 4.69^a^	9.06 ± 2.49^ab^
AHC10	86.82 ± 6.48^c^	90.65 ± 7.28^d^	25.46 ± 4.38^c^
AHC12	41.42 ± 4.20^a^	74.44 ± 4.36^c^	5.10 ± 1.37^a^
AHC21	35.41 ± 4.86^a^	62.31 ± 6.40^b^	4.14 ± 1.21^a^
AHC24	68.67 ± 3.19^b^	83.64 ± 6.90^cd^	35.01 ± 5.86^d^
AHC28	64.65 ± 6.69^b^	78.58 ± 5.36^cd^	20.40 ± 4.08^c^
AHC32	62.06 ± 8.65^b^	74.70 ± 5.21^c^	5.24 ± 1.84^a^
BB-12	80.24 ± 7.99^c^	87.19 ± 6.65^cd^	14.48 ± 4.01^b^

Results are expressed as the mean ± SD;^a-d^, different letters along the column represent statistical significance (*P*< 0.05).

### Antibacterial activity

Four *Bifidobacterium* isolates AHC3, AHC10, AHC24 and AHC28 were tested for their antibacterial activities against 4 pathogenic strains, including *E*. *coli*, *S*. *aureus*, *S*. *Enterica* and *S*. *Sonnei*, and the results were indicated in Table 5. All the 4 isolates had bacteriostatic effects on *S*. *aureus*. Among them, the antibacterial activity of AHC3 reached to 16.1 ± 2.4 mm, significantly higher than that of the reference strain BB-12 (11.1 ± 3.4) (*P*<0.05). All the 4 isolates showed bacteriostatic abilities on *E*. *coli* except AHC10. Three isolates exhibited inhibitory effects on *S*. *enterica*, including AHC3, AHC10 and AHC28. Out of the 4 isolates, only 2 isolates displayed inhibitory effects on *S*. *sonnei*, and no antibacterial abilities of AHC24 and AHC28 was observed on *S*. *sonnei*. Noticeably, among the 4 isolates, only AHC3 possessed antibacterial activities against the 4 tested pathogenic strains which had a broad spectrum of inhibition in our research, it was reasonable to choose AHC3 for the next step of safety test.

**Table 5:** Antibacterial activities of 4 isolated *Bifidobacterium* strains.

Strains	Antibacterial activity (mm)			
	*E*. *coli*	*S*. *aureus*	*S*. *enterica*	*S*. *sonnei*
AHC3	11.7 ± 3.0^a^	16.1 ± 2.4^b^	10.3 ± 1.9^a^	9.5 ± 2.0^a^
AHC10	-	12.5 ± 1.5^ab^	11.2 ± 1.7^a^	8.6 ± 1.4^a^
AHC24	10.7 ± 1.9^a^	8.8 ± 1.7^a^	-	-
AHC28	7.9 ± 0.8^a^	8.4 ± 1.9^a^	8.5 ± 1.5^a^	-
BB-12	17.2 ± 3.1^b^	11.1 ± 3.4^a^	16.7 ± 2.1^b^	10.0 ± 1.5^a^

Results are expressed as the mean ± SD;^a-b^, different letters along the columnrepresent statistical significance (*P*< 0.05); -, no antibacterial activity detected.

### Safety of *Bifidobacterium* strain AHC3

**Fig 2:**
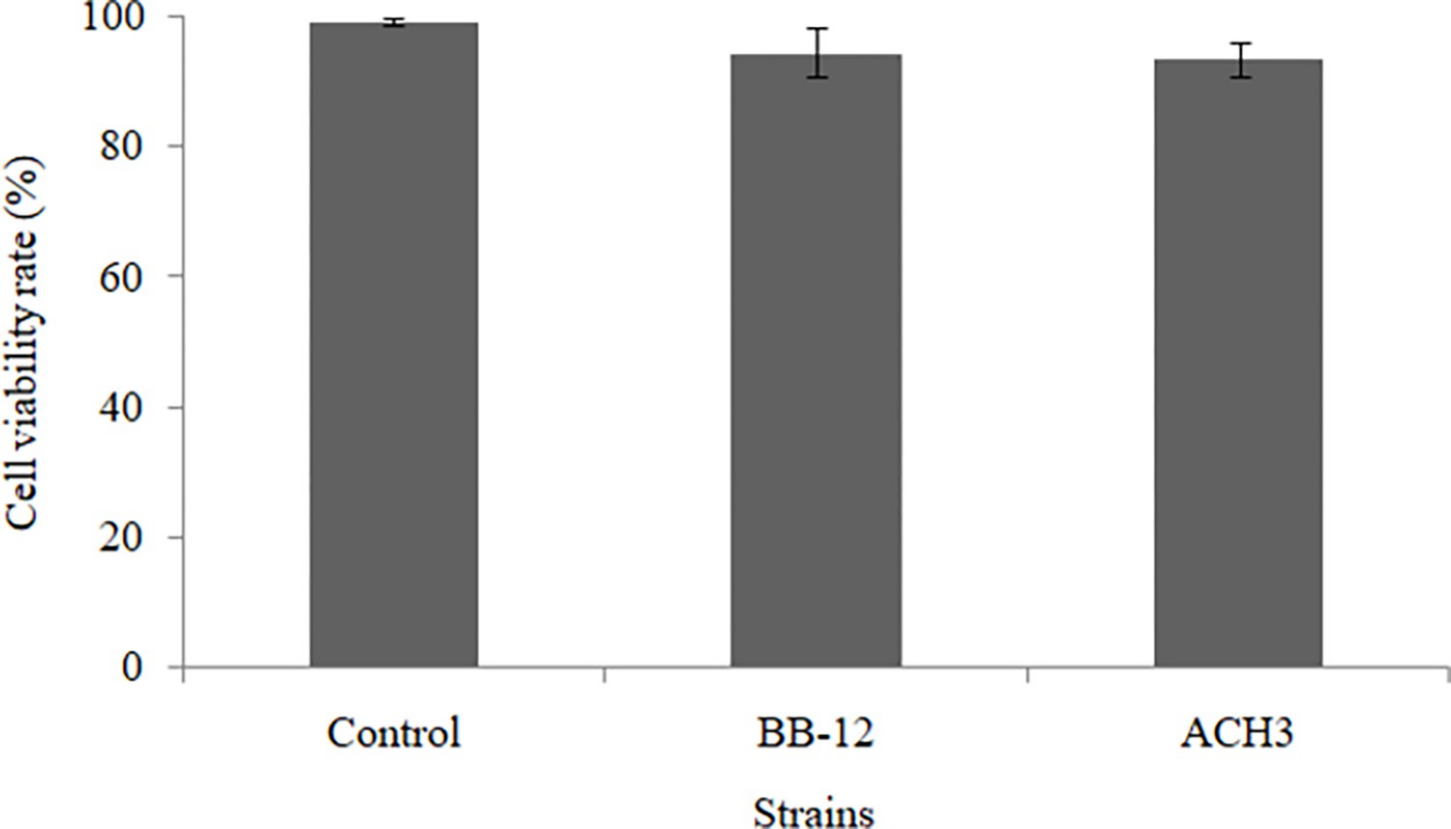
Cytotoxicity assay of *Bifidobacterium* strain AHC3 on HT-29 cell line. Error bars represent deviations of mean values (n = 3).

The hemolysis, biogenic amines production and antibiotic sensitivity of isolated *Bifidobacterium* strain AHC3 were investigated, and the results were presented in Table 6. No hydrolytic circle and color change observed confirmed that AHC3 had absence of hemolytic activity and production of biogenic amines, respectively. Out of 12 commonly used antibiotics, AHC3 was sensitive to 8 kinds of antibiotics, including Gentamicin, Penicillin, Chloroamphenicol, Rifampicin, Erythromycin, Clindamycin, Ciprofloxacin and Ampicillin. On the contrary, it was resistant to Vancomycin, Kanamycin, Streptomycin and Tetracycline. MTT method was employed to determine the cytotoxicity of AHC3 to HT-29 cell line, and the result was shown in Fig 2. The survival rate of HT-29 cells did not declined significantly, but remained at a high level (93.61 ± 2.61%). Compared with the reference strain BB-12 (94.47 ± 3.76%), no significant difference (*P*<0.05) was observed, suggesting that AHC3 was lack of cytotoxicity to HT-29 cell line.

**Table 6:** Antibiotic sensitivity, hemolysis and biogenic amines production of *Bifidobacterium*strain AHC3.

	AHC3	BB-12
**Antibiotics sensitivity**		
Vancomycin (30 μg)	R	S
Gentamicin (10 μg)	S	R
Penicillin (10 μg)	S	S
Chloramphenicol (30 μg)	S	S
Kanamycin (30 μg)	R	R
Streptomycin (10 μg)	R	R
Rifampicin (5 μg)	S	S
Tetracycline (30 μg)	R	S
Erythromycin (15 μg)	S	S
Clindamycin (2 μg)	S	S
Ciprofloxacin (5 μg)	S	R
Ampicillin (10 μg)	S	S
**Hemolysis**	-	-
**Biogenic amines production**	-	-

R, resistant to antibiotics. S: sensitive to antibiotics; -, No hemolysis or production of biogenic amines.

### SEM imaging on AHC3

The morphological characteristic of AHC3 strain under scanning electron microscope was seen in Fig 1C and 1D. Under SEM imaging, AHC3 strain was rough on the surface and short rod or ellipsoid.

### 16S rRNA gene sequencing and identification

**Fig 3:**
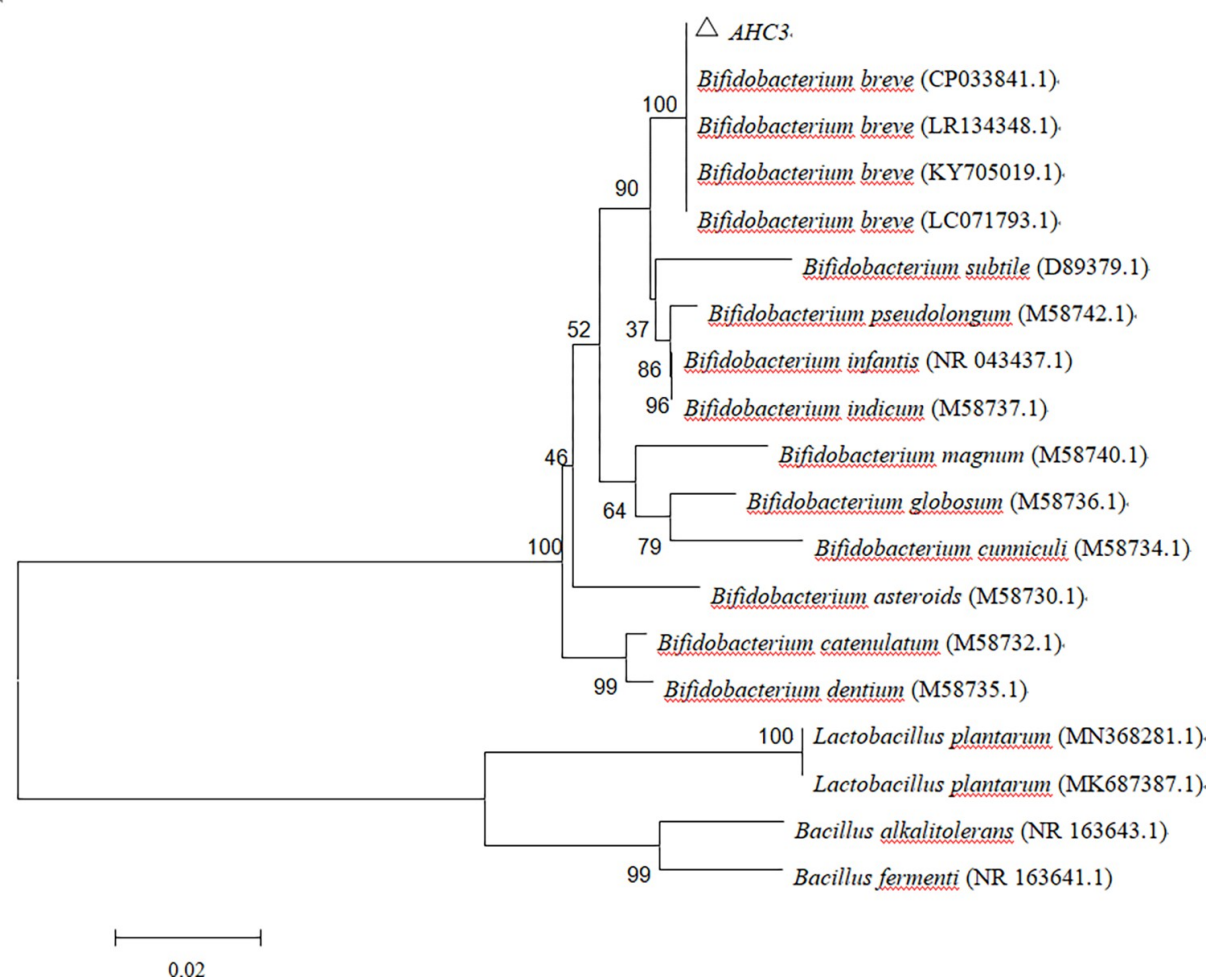
Phylogenetic tree constructed based on 16S rRNA gene sequence ofAHC3.

The 16S rRNA gene of AHC3 was sequenced and identified. The 16S rRNA gene sequencing results of AHC3 were uploaded to NCBI (the accession No. MN 736535), and BLAST was employed for sequence similarity analysis. The phylogenetic tree constructed of AHC3 was displayed in Fig 3. The homology of AHC3 and *Bifidobacterium breve* 16S rRNA gene sequence was up to 100%. Consequently, according to the 16S rRNA gene sequencing and the results of the above physiological and biochemical preliminary identification, AHC3 strain was identified as *Bifidobacterium breve* and simply named *B*. *breve*AHC3.

### Pathological evaluation of intestinal tissue in NEC model

**Fig 4:**
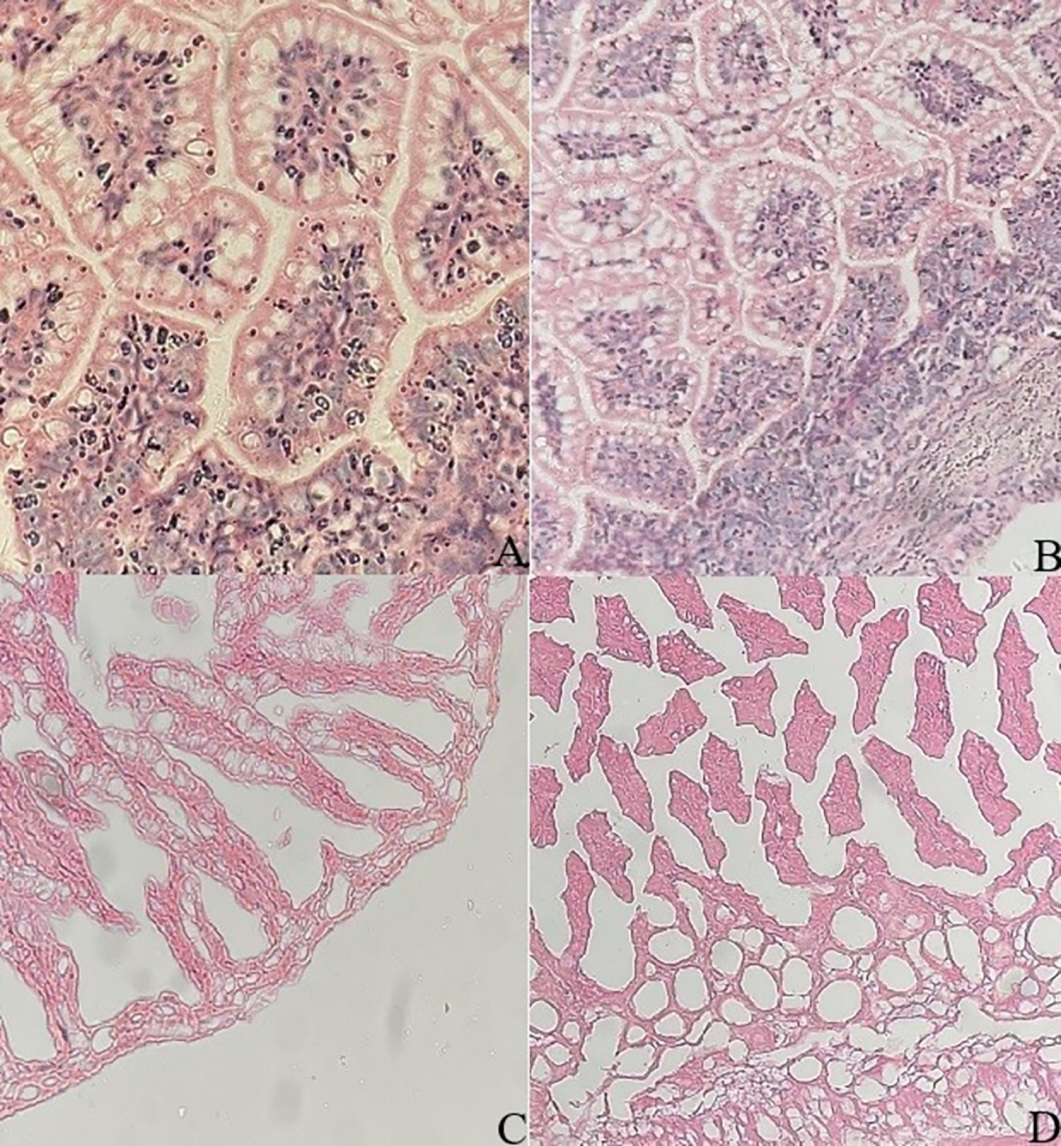
Representative images of pathological features in intestinal tissue from neonatal rat model of NECwith H&Estaining. (A) Blank control group; (B) *Bifidobacterium* control group; (C) NEC model group; (D) *Bifidobacterium* intervention group; Magnification, ×200.

Light microscopic images of pathological features of intestinal tissue sections from neonatal SD rats in group A, B, C and D after H&E staining were shown in Fig 4. After modeling, severe separation of submucosa or lamina propria, disintegration, separation, shedding and loss of intestinal villi glands were observed under light microscope in group C. In contrast, after modeling and *B*. *breve* AHC3 gavage in group D, the histopathologic changes were milder than those in group C, and slight edema of submucosa and muscle layer, moderate separation of submucosa and lamina propria, and existence of intestinal villi structure were observed. Intestinal villi in group A and group B were intact, and there was no significant difference between the two groups.

### Intestinal histological scoreof intestinal tissue in NEC model

**Fig 5:**
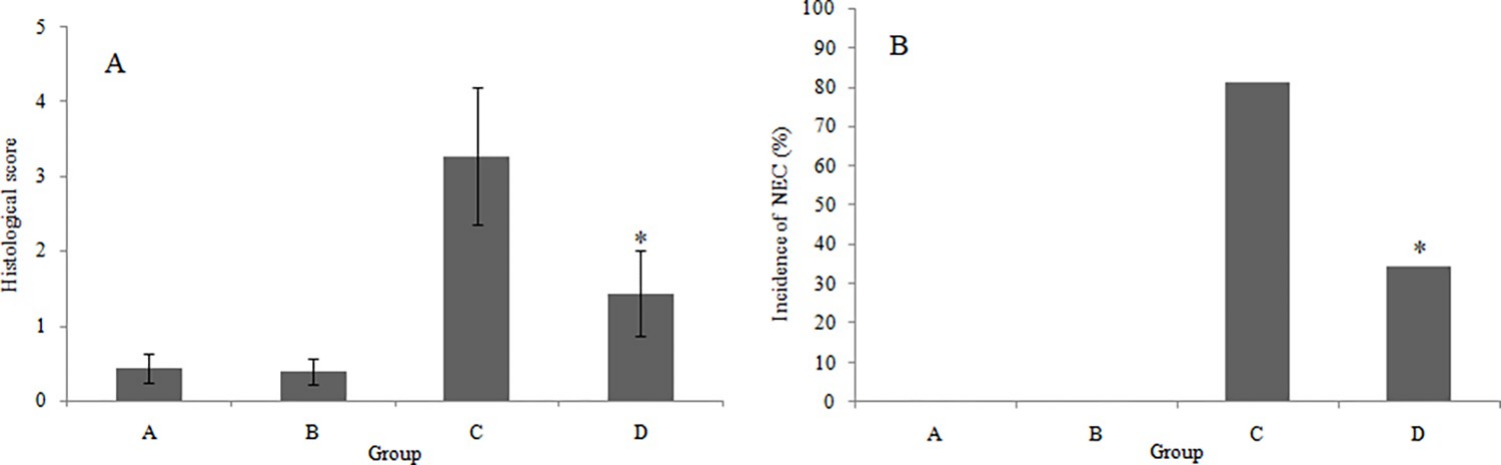
Intestinal histological score and incidence of NEC in the neonatal rat model of NEC. (A) Intestinal histological score of 4 groups;Error bars represent deviations of mean values (n = 32); *P< 0.05 vs the NEC group;(B) Incidence of NEC in 4 groups;*P< 0.05 vs the NEC group.

The intestinal histological scores of 4 groups were illustrated in Fig 5A. Among the 4 groups, the intestinal histological score in group C was the highest (3.271±0.914), which was significantly higher than that in group A (0.438±0.260) (*P*<0.05). Compared with group C, the intestinal histological score of group D significantly declined with the score of 1.438±0.571 (*P*<0.05), while no significant difference was observed between group A and group B (*P*>0.05). In addition, the incidence of NEC in each group waspresentedin Fig 5B. The incidence rates of NEC in group A, B, C and D were 0% (0/32), 0% (0/32), 81.25% (26/32) and 34.38% (11/32), respectively. Compared with group C, the incidence rate of NEC in group D significantlydecreased (*P*<0.05).

### Evaluation of inflammatory response in the intestinal tissue of NEC model

In order to investigate the effect of *B*. *breve* AHC3 on the inflammatory response in NEC model, the inflammatory cytokines in the intestinal homogenate of 4 groups were detected by ELISA, including proinflammatory factor TNF-α and antiinflammatory cytokine IL-10. The results were shown in Table 7. In the 4 groups, the level of TNF-α in group C was the highest, reaching up to 70.968±2.538pg/mL, which was significantly higher than that in group A (*P*<0.05). After the intervention of *B*. *breve* AHC3, the level of TNF-α (41.086±1.134pg/mL) in group D declined,which was significantly lower than that in group C (*P*< 0.05). Contrary to the results of TNF-α, the level of IL-10 in group D (150.817±10.106pg/mL) was significantly higher than that in group C (79.935±11.143pg/mL) (*P*< 0.05). No significant difference in the levels of TNF-α and IL-10 was observed between group A and group B.

**Table 7:** Levels of inflammatory cytokine and gray value of iNOS in the intestinal tissue of neonatal rat model of NEC.

Group	N	TNF-α (pg/mL)	IL-10 (pg/mL)	Gray value of iNOS
A	32	29.877±3.489^a^	182.197±10.480^c^	85.730±9.637^a^
B	32	24.992±3.286^a^	190.531±14.553^c^	84.738±12.274^a^
C	32	70.968±2.538^c^	79.935±11.143^a^	133.864±23.297^b^
D	32	41.086±1.134^b^	150.817±10.106^b^	95.958±11.221^a^

Results are expressed as the mean ± SD;^a-c^, different letters along the column represent statistical significance (*P*< 0.05).

### Evaluation of iNOSexpressionin intestinal tissue of NEC model

**Fig 6:**
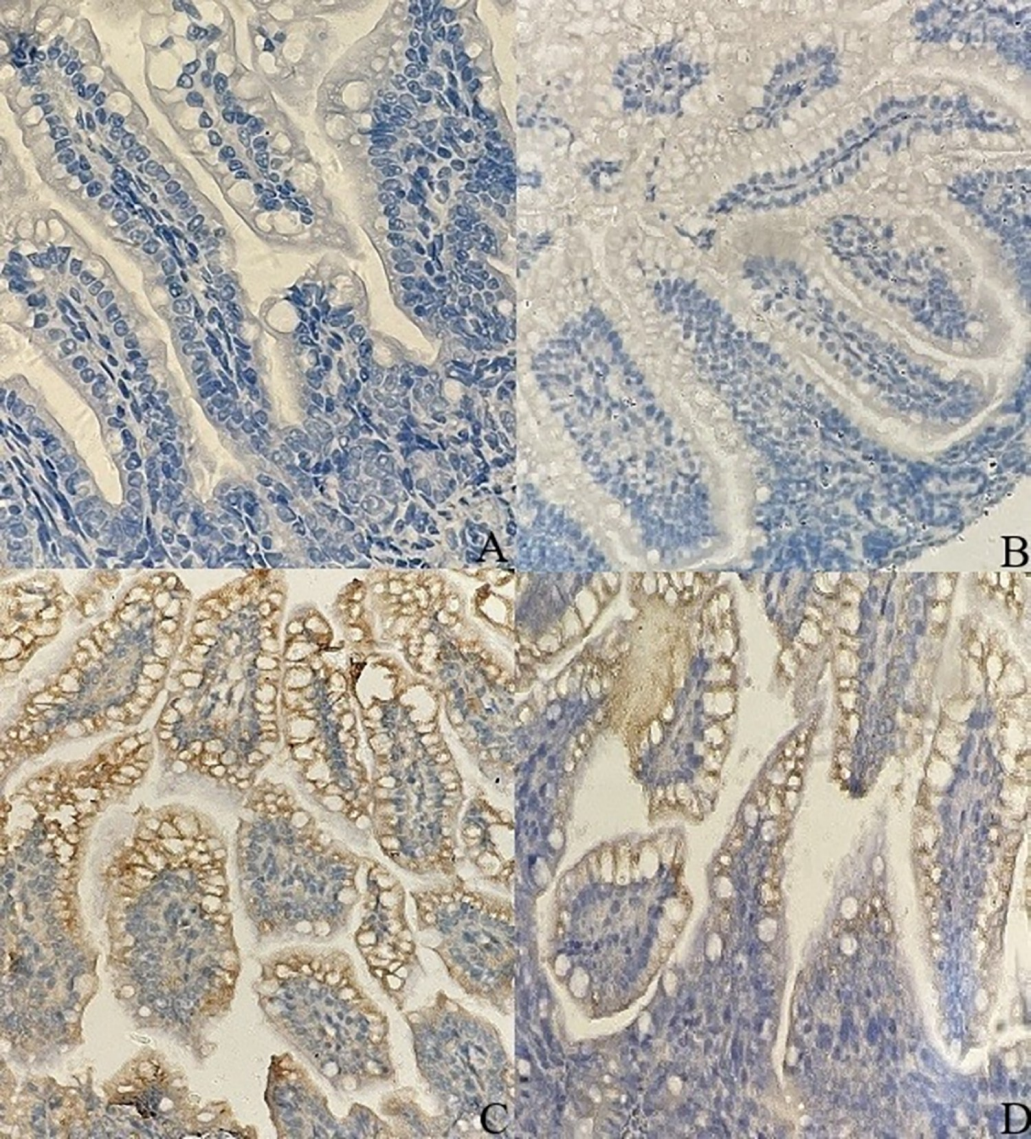
Representative images of iNOS expression in intestinal tissuefrom neonatal rat model of NEC with immunohistochemical staining. (A) Blank control group; (B) *Bifidobacterium* control group; (C) NEC model group; (D) *Bifidobacterium* intervention group; Magnification, ×400.

The iNOSexpression in intestinal tissues was determined by immunohistochemical staining. The iNOS gray values in intestinal tissues of the 4 groups were indicated in Table 7. Compared with the iNOS gray value in group A (85.730±9.637), after NEC modeling, the iNOS gray value in group C (133.864±23.297) significantlyincreased (*P*<0.05). After the intervention of *B*. *breve* AHC3, the iNOS gray value in group D (95.958±11.221) was significantly lower than that in group C (*P*<0.05). No significant difference was observed between group A and group B. The light microscopic observation results of immunohistochemical staining were consistent with the iNOS gray value. The iNOS immunostaining of intestinal tissuein group A and B were the lowest (Fig 6A and 6B), and the number of positive particles and staining intensity in group C (Fig 6C) were significantly higher than those in group A and B. However, after *B*. *breve* AHC3intervention, the iNOS expression in intestinal tissue cells in group D was noticeably suppressed (Fig 6D).

## Discussion

Tolerance test to artificial gastrointestinal conditions is one of the necessary conditions for screening potential probiotic candidate [54]. Sim et al. [55] reported the research on thetolerance to artificial simulated gastrointestinal juices of *E*. *faecium* L11 isolated from chicken cecum, and the viability of *E*. *faecium* L11 was 66.8 ± 3.3% and 62.8 ± 5.9% respectively after the treatment of artificial simulated gastric juice and intestinal juice, which was lower than the results of our study. Similar to the results of the present work, 4 selected *Bifidobacterium* strains were cultured in artificial simulated gastric juice at pH 2.0 for 3 h, the survival rates of all the 4 strains exceeded 80%, and the survival rate of *B*. *pseudocatenulatum* IMAUFB084 reached to 85.98% [56]. Liu et al. [57] concluded that among 6 *Bifidobacterium* spp. strains, *B*. *breve* A03, *B*. *Breve* A04 and *B*. *adolescentis* L-1 showed better tolerance to artificial gastrointestinal juices, and their survival rates were 87.8%, 89.9% and 89.4% respectively, which was superior to the results of this study. In the present research, the tolerance of individual *Bifidobacterium* strain to artificial simulated gastrointestinal conditions varied among strains. Compared with the other 7 strains, 14 strains had significantly higher tolerance to artificial gastrointestinal conditions, and the final survival rates exceeded 50%, suggesting that the survival ability of *Bifidobacterium* stain in artificial simulated gastrointestinal conditions is strain-specific.

In order to investigate the probiotic potential of *Bifidobacterium* strains, it is necessary to assay the adhesion ability one by one [58]. In addition, the HT-29 cell line has been usually employed as a model system simulating the adhesion to human intestinal epithelial cells for *in vitro* method to evaluate the adhesion ability of probiotics [59, 60]. It was reported that*B*. *dentium* N8 showed higher adhesion rate to HT-29 cells with 55.64%, indicating that it possessed the preeminent adhesion ability [61]. Jang et al. [62] used HT-29 cell line to assay the adhesion ability to intestinal mucosa, which showed that *B*. *longum* subsp. *longum*CACC517 strain had the highest adhesion activity to HT-29 cell line (61.7%). Other study also revealed that *B*.*animalis* ssp. *Lactis* UABla-12 showed the adhesion rate of 79–86% to HT-29 cells [63]. In our research, 8 of the 14 *Bifidobacterium* isolates exhibited high adhesion to HT-29 cell line, and the adhesion capabilities of AHC12 and AHC28 were comparable to the reference strain BB-12. The aggregation and hydrophobicity phenotypes may be involved in adhesion, but they are not the unique mechanisms related to adhesion. Additionally, the moonlighting protein transaldolase has been proved to play a part in the adhesion of *Bifidobacterium* [59, 64].

*Bifidobacterium* possesses strain-specific antioxidant property and reduces oxidative damage [65]. DPPH, ABTS and superoxide anion radical scavenging, as three frequently-used experimental methods, are currently used to assay the antioxidant property of probiotics [58, 66]. In the present research, 4 isolates of AHC24, AHC10, AHC3 and AHC28 had respectable DPPH, ABTS and superoxide anion radical scavenging capacities. Our results were consistent with other studies on the antioxidant property of *Bifidobacterium*.*B*. *longum* BL-10 isolated from healthy baby feces by Dong et al. [67], exhibited better antioxidant performance than *B*. *animalis*subsp. *lactis* BB-12.It was illustrated by Yu et al. that the scavenging activities of *B*. *pseudolongum* YY-26 on DPPH radical and superoxide anion radical were 97.58% and 43.13%, respectively [68]. Kang et al. [69] also confirmed that *B*. *bifidum* MG731 showed the highest DPPH radical scavenging activity (90.6%), and *B*. *lactis* MG741 displayed the highest ABTS radical scavenging activity (99.5%). The results of the above two studies were better than those of this study. The antioxidant property of *Bifidobacterium* may be related to a variety of antioxidant compounds, such as soluble proteins and exopolysaccharides [70, 71]. They may also play a role through specific molecular mechanisms in charge of barrier against the oxidative stress [72]. Each antioxidant substance has its own special mechanism of action, resulting in species- and strain-specific antioxidant property [73].

The determination of antimicrobial activity is a necessary step in the screening process of *Bifidobacterium* [74, 75]. Aalipanah et al. [37] used disk diffusion method to evaluate the antibacterial property of *B*. *bifidum* strain originated from chicken intestinal tract, which had antibacterial property against *E*. *coli*. Jang et al. [62] reported that *B*. *longum* CACC517 isolated from dog feces showed antibacterial activity against *S*. *enteritidis* and *E*. *coli*. Cai et al. [76] also revealed that 14 representative *Bifidobacterium* strains isolated from infant feces in the Ughur population of northwestern China exhibited strong antibacterial activities. Among the 14 strains investigated,*B*. *longum* subsp. *infantis* (BF48-2, BF17-4, BF67-13) and *B*. *bifidum* (BF87-11, BF52-1) inhibited*E*. *coli*, *S*. *enterica* and *S*. *aureus*, with a wide spectrum inhibitory effects. The results of the current work were basically consistent with those of the above studies. Among the 4 isolates, only AHC3 possessed antibacterial activities against the 4 tested pathogenic strains. It was indicated that AHC3 had respectable antibacterial activity. The antibacterial activity of *Bifidobacterium* may be due to the organic acids such as acetic acid, propionic acid, phenyllactic acid and free fatty acids, and protein compounds including bacteriocin and ablatin produced during the metabolism of *Bifidobacterium* [77, 78].

As the main virulence factor, hemolysis is a special feature of pathogen. The selected *Bifidobacterium* strains must be absence of hemolytic activity to be safely used in the host body [79]. In the present research, no hemolysis of *B*. *breve*AHC3 was observed, which was consistent with the research results of Cizeikiene and Jagelaviciute [65].Biogenic amines are a kind of low molecular mass organic based with aromatic and heterocyclic structures, which is harmful to human health [80]. In this study, no biogenic amines production of *B*. *breve*AHC3 was observed. It was consistent with the research results of Ku et al. [25] on the biogenic amines production by *Bifidobacterium*.The isolates may carry antibiotic resistance genes, which could be transmitted to pathogens through horizontal gene transfer, resulting in the emergence of antibiotic resistant pathogens [79]. The result of our work was consistent with the studies on antibiotic sensitivity of *Bifidobacterium*. Choi et al. [81] reported that *B*. *breve* IDCC4401 isolated from infant feces showed antibiotic resistance to vancomycin. Zuo et al. [59] found that *B*. *breve* IF2-173 and IF2-174 had resistance to Kanamycin and Streptomycin. Kim et al. [79] also observed that *B*. *breve* MG729 was resistant to Tetracycline.*Bifidobacterium* is generally resistant to Gentamicin, Kanamycin and Streptomycin [82]. The genetic basis of these drug resistances is chromosome mutation, so they are unlikely to transfer to other bacteria [83]. It was reported that a fecal isolate of *B*. *breve* NCIM 5671 exhibited no indication of any cytotoxicity, and the viability of HT-29 cells was not affected, with a survival rate of 94.78 ± 2.27% [84]. The results of our research were consistent with the above results. It was indicated that *B*. *breve*AHC3 had no cytotoxicity to HT-29 cell line.

Although great progress has been made in the treatment of NEC in recent years, there is still no cure for this disease. Studies have shown that probiotics are the most promising intervention to prevent NECat present [85, 86]. However, differences in the protective capacity of the strains against NEC rat models were found in a recent comparison of some *Bifidobacterium* and *Lactobacillus* strains [87]. Selection of appropriate probiotic strains seems to be the key to achieving the protective effect on NEC. It was illustrated that administration of live *B*. *breve*AHC3 significantly reduced not only the incidence of NEC (from 81.25% to 34.38%) but also the severity of ileal injury in the present research, suggesting that *B*. *breve*AHC3might have the potential in the treatment of NEC. Similar to our results, Khailova et al. [13] reported that oral administration of *Bifidobacterium* could reduce the severity and incidence of NEC. Compared with NEC group, the rats had significantly lower ileal injury and the incidence of NEC in *Bifidobacterium* intervention group.Underwood et al. [31] also observed that administration of *B*. *longum*subsp.*infantis* had protective effect on neonatal rat model of NEC, which was characterized by a decrease in NEC incidence and histological score. It was found by Zhou et al. [27] that *Bifidobacterium* had a protective effect on the intestinal tract of newborn rats suffering from NEC, it could reduce the severity of NEC and intestinal injury. Probiotics added into the intestine may help to reduce the risk of NEC, but the exact mechanism is still unclear. There may be multiple mechanisms to protect the intestine, including stimulating mucosal immune defense response, enhancing the integrity of mucosal barrier, increasing the production of secretory IgA, promoting the development and maturation of intestinal immune function, and diversifying intestinal microbiota, etc. [88].

Inflammation, as an innate immune response, is a basic self-protective response in the human body [89]. However, due to the immaturity of intestine and immune system, neonates are prone to excessive inflammation, which may be a key factor in the pathogenesis of NEC [17]. A number of different proinflammatory and antiinflammatory factors have been proved to be associated with NEC. In NEC model, some probiotics have been identificated to inhibit the inflammatory reaction to maintain the intestinal barrier function. Xiang et al. [51] found that compared with NEC group, *Bifidobacterium* could significantly reduce the secretion of TNF-αand alleviate the injury of NEC by inhibiting the release of inflammatory cytokines in *Bifidobacterium* intervention group. Lueschow et al. [90] reported that *B*. *infantis* EVC001 significantly reduced the level of inflammatory cytokines.It was documented that *B*. *fragilis* strain ZY-312ZY-312 not only reduced the expression of TNF-α, but also enhanced the expression of IL-10, thus effectively alleviating inflammation and preventing the development of NEC [91]. The results of our research were similar to those of the above studies. In this study, after *B*.*breve*AHC3 intervention, it was indicated that *B*.*breve*AHC3 could effectively reduce the level of proinflammatory factor TNF-α and increase the level of antiinflammatory factor IL-10, which had anti-inflammatory potential in the intestinal tract of NEC rats. The mechanism of protective effect on developing intestine from probiotics has not been fully understood. The application of probiotics (such as *Bifidobacterium*) may inhibit the growth of pathogenic bacteria and reduce the intestinal production of proinflammatory cytokines by reducing the pH value in intestinal cavity and increasing the production of antibacterial substances [92, 93].

iNOS is one of the influential enzymes responsible for NEC process [94]. iNOSis transcribed and translated under the stimulation of LPS and some cytokines. Once induced, it continuously synthesizes NO. The continuous release of NO may lead to intestinal cellular damage and intestinal barrier failure [22]. At the same time, excessive NO activates and amplifies immune and inflammatory reactions, and these proinflammatory factors in turn up-regulate the expression of iNOS, resulting in aggravating epithelial cell damage [95]. The up-regulation of iNOSsimultaneously inhibits the intrinsic repair mechanism, such as intestinal cell migration and proliferation. The imbalance between epithelial cell damage and repairsresults in the persistent mucosal defects, which further aggravates intestinal injury and enterocyte apoptosis of NEC [96]. Application of probiotics reduces the expression of iNOS during NEC, so as to alleviate the injury of NEC. Hunter et al. [97] reported that *Lactobacillus* decreased intestinal epithelial damage in experimental NEC by reducing iNOS production and maintaining epithelial integrity. Underwood et al. [31] also proved that administration of *B*. *longum* subsp. *infantis* significantly reduced the expression of iNOS. The results of the present research were consistent with the above results. Through the intervention of *B*.*breve*AHC3, the gray value of iNOS and expression of iNOS in intestinal tissue of NEC rats was significantly reduced and inhibited, respectively. This may be partially attributed to the reduction of TNF-α level by the intervention of*B*.*breve*AHC3, as the proinflammatory cytokine TNF-αis usually considered as a strong inducer of iNOS expression. In addition, exogenous supplementation of *Bifidobacterium* contributes to prompting the immune system to play an active role in eliminating oxygen free radicals, inhibiting the production of lipid peroxides, protecting enterocellular organizational structure and maintaining the role of mucosal barrier [98, 99].

## Conclusions

To sum up, the *B*. *breve* AHC3 isolated from the chicken intestines not only exhibited excellent probiotic potential, including high tolerance to artificial simulated gastric conditions, strong adhesion to HT-29 cells, outstanding antioxidant capacity and broad-spectrum inhibition on 4 pathogenic strains, but also possessed reliable safety, such as lack of hemolysis, no biogenic amine production, absence of cytotoxicity, and normal antibiotic sensitivity in the present research. Additionlly, in the neonatal SD rat model of NEC, through reducing the inflammatory reaction in the ileum and inhibiting the expression of iNOS in intestinal tissue cells, *B*. *breve* AHC3 had an available protective effect on intestinal injury of NEC. *B*. *breve* AHC3 may be a promising candidate strain with probiotic function for the treatment of human NEC.

## Supporting information

S1 Table Tolerance to artificial simulated gastrointestinal conditions. (XLSX)

S2 Table Adhesion capacity to HT-29 cell. (XLSX)

S3 Table Antioxidant capacities. (XLSX)

S4 Table Antibacterial activity. (XLSX)

S5 Table Cytotoxic activity. (XLSX)

S6 Table Intestinal histological score. (XLSX)

S7 Table Levels of inflammatory cytokine. (XLSX)

S8 Table Gray value of iNOS. (XLSX)
